# Effect of Graphene Nanowall Size on the Interfacial Strength of Carbon Fiber Reinforced Composites

**DOI:** 10.3390/nano8060414

**Published:** 2018-06-07

**Authors:** Xiao Wang, Chaolong Li, Yao Chi, Mingxing Piao, Jin Chu, Heng Zhang, Zhenghao Li, Wei Wei

**Affiliations:** 1 Key Laboratory of Optoelectronic Technology & Systems, Ministry of Education of China, College of Optoelectronic Engineering, Chongqing University, Chongqing 400044, China; wangxiao@cigit.ac.cn (X.W.); lizhenghao@cqu.edu.cn (Z.L.); 2 Key Laboratory of Multi-Scale Manufacturing Technology, Chongqing Institute of Green and Intelligent Technology, Chinese Academy of Sciences, Chongqing 400714, China; lichaolong@cigit.ac.cn (C.L.); piaomx@cigit.ac.cn (M.P.); chujin@cigit.ac.cn (J.C.); zhangheng@cigit.ac.cn (H.Z.)

**Keywords:** graphene nanowalls, carbon fiber, interface, interfacial shear strength, interlaminar shear strength, chemical vapor deposition

## Abstract

Graphene nanowalls (GNWs) with different sizes (i.e., length and height) were grown directly on the surface of individual carbon fibers (CFs) using a radio frequency plasma-enhanced chemical vapor deposition (RF-PECVD) technique. The size was controlled by varying the deposition time. The GNW-modified CFs were embedded into epoxy resin matrix to prepare a series of carbon-fiber-reinforced composites (CFRCs). The results indicated that GNWs were remarkably effective in improving the interfacial shear strength (IFSS) and interlaminar shear strength (ILSS) of the carbon-fiber-reinforced composites. The enhancement effect on the strength strongly depended on the size of GNWs. It increased with the increase in the GNWs’ size and reached the maximum upon the incorporation of GNWs that were grown for 45 min. Noticeable increases of 222.8% and 41.1% were observed in IFSS and ILSS, respectively. The enhancement mechanism was revealed by means of scanning electron microscope (SEM) fractography analysis. However, further increase of GNW size led to no more improvement in the shear strength. It could result from the increased defect concentration and wrinkle size in the GNWs, which deteriorated the strength.

## 1. Introduction 

Nowadays, carbon-fiber-reinforced composites (CFRCs) have attracted increasing attention due to their high strength, stiffness, and toughness [1]. The performance of the composites depends not only on the properties of carbon fiber (CF), but also on the fiber-matrix interface [2,3,4,5]. Good interfacial interaction enables effective load transfer between the CF and the matrix, which significantly promotes the strength of the composites. However, pristine carbon fiber is essentially microcrystalline graphite material, which has smooth surface, strong chemical inertness, and poor wettability [6]. It shows weak interfacial adhesion with the matrix. The reinforcement efficiency is limited [7]. In general, interfacial interaction, wettability, and defects concentration are the main factors affecting the effectiveness of the CF reinforcement process, since they are closely related to the load transfer efficiency and stress concentration factor that determine the interfacial strength and toughness. Therefore, it is vitally important to improve the interface in the development of high performance CFRC. 

The surface modification of CFs by means of chemical or physical treatment has been one of the typical approaches to enhancing the interface of CFRC. The modified CFs possess active functional groups and increased surface area, which could contribute to the formation of strong and stable interaction between the CFs and the matrix. Recently, nanomaterials such as carbon nanotubes (CNTs) [8,9,10,11,12], ZnO nanowires [13], nano-whiskers [14], and graphene oxide (GO) [15,16,17] have been utilized to modify the CFs in composites. The hybrid micro/nanocomposites combine the potential benefits of nanoscale reinforcement with the well-established fibrous composites. The interfacial shear strength (IFSS) and interlaminar shear strength (ILSS) of the CFRC are enhanced after the incorporation of these nanomaterials. The growth of graphene nanowalls (GNWs) and similar structures on carbon fibers by means of hot filament chemical vapor deposition (HFCVD) and plasma-enhanced chemical vapor deposition (PECVD) has also been reported [18,19,20]. In our previous report [21], GNWs were successfully grown on the surface of carbon fibers using radio frequency PECVD (RF-PECVD) methods. The GNWs stacked on the surface of CFs via strong interactions. The presence of GNWs acted as a stable interface providing a ‘tenon-mortise’-like mechanical interlock between the CFs and matrix in a large area. The interfacial shear strength of the composite significantly increased compared to other nanomaterials-incorporated CFRC systems. However, the in-depth enhancement mechanism was not sufficiently understood. The size effect of the nanowalls on the properties was not clear. 

In this study, a series of GNWs with different sizes, including length and height, were grown on CFs. The relationship between the growth time and GNW size was revealed. Moreover, the effect of the GNW size on the interfacial strength of the CFRC was investigated. The understanding of the reinforcing mechanism was also developed. We attempt to provide a theoretical support for the design of high performance CFRC materials. 

## 2. Experimental Section

### 2.1. Materials

Carbon fibers (M55j) (Tokyo, Japan), with an average diameter of 5 μm, were purchased from Japan Toray. Epoxy resin (TS190) and its curing agent (TS195) were supplied by Dow Chemical Compounds (Chongqing, China). H_2_ and CH_4_ were purchased from Chongqing Ruixin Gas Co., Ltd. (Chongqing, China). Acetone was supplied by Chongqing Chuandong Chemical Co., Ltd. (Chongqing, China)

### 2.2. Graphene Nanowalls (GNWs) Growth on Carbon Fiber (CF)

GNWs were grown on carbon fibers using a tube-style RF-PECVD system with a glow discharge plasmas apparatus (BEO BTF-1200C GMF-3Z, Anhui, China) In brief, after removing sizing agent of carbon fibers with acetone reflux method, the fibers were placed into the center of a quartz tube. H_2_ and CH_4_ were supplied with a ratio of 1:1 and the radio frequency (RF) power was set at 250 W. The GNWs were grown at 750 °C for 0, 15, 30, 45, 60, and 90 min, respectively. The resultant carbon fibers were denoted as CF0, CF1, CF2, CF3, CF4, and CF5. 

### 2.3. Sample Preparation for Single-Fiber Segmentation Test 

Single-fiber reinforced composite samples were prepared by suspending a single filament of CF along the axis of a Teflon dogbone mold (ISO 527-2/1BA standard [22]). The epoxy resin and curing agent were mixed (weight ratio of 3:1) and poured into the mold followed by curing reaction at 60 °C for 1 h and post-cure at 80 °C for 1 h. The specimens of the CFRC embedded with different single fibers were defined as SCF0-RC, SCF1-RC, SCF2-RC, SCF3-RC, SCF4-RC, and SCF5-RC, respectively. 

### 2.4. Sample Preparation for Three-Point Short Beam Shear Test 

Composite laminates were prepared for three-point short beam shear test. In the manufacturing process, CFs were impregnated with the epoxy resin and curing agent at a weight ratio of 3:1 to prepare prepregs. The prepreg sheets were laid up in the unidirectional fiber orientation and cured in a vacuum bag at 60 °C for 1 h followed by post-cure at 80 °C for 1 h. The fiber volume fraction in the composite laminates was about 50%. The composites containing different types of CFs were defined as CF0-RC, CF1-RC, CF2-RC, CF3-RC, CF4-RC, and CF5-RC, respectively.

### 2.5. Characterization

A field emission scanning electron microscope (FESEM, JSM-7800F, JEOL (Tokyo, Japan)) was used to observe the GNW-modified CFs and their composites (operated at 10 kV). The length and height of the GNWs were measured based on the surface and cross-sectional SEM images of the CFs, respectively, using ImageJ (Bethesda, MD, USA). At least two hundred GNWs were measured to obtain the average values for each CF type. An atomic force microscope (AFM, Dimension Edge, Bruker (Billerica, MA, USA)) was used to observe the surface profiles of the fibers using tapping mode. The scanning rate was 0.5 Hz and the scanning scope was 3 μm × 3 μm. The surface roughness, Ra, that arose from the GNWs was measured along the fibers’ longitudinal direction to avoid the influences of the grooves and the curved surface of the fibers. Raman spectra were determined via Raman spectrometer (Renishaw inVia ManualWiRE3.4 (London, UK)) with 532 nm laser. 

The single-fiber tensile test was performed using the LLY-06ED single-fiber strength testing machine (Shandong, China), according to the ASTM D3379 standard [23]. Before testing, each fiber filament was bonded firmly to a paper mounting with slot of length 20 mm. The mounting was clamped in the grips, and the fiber was aligned with the loading axis. Both sides of the mounting were cut at mid-gauge before applying the load. The test was conducted at room temperature with a crosshead speed of 10 mm/min. The tensile strength, *σ_f_*, of the fiber was calculated according to the equation [23]:

(1) $$\sigma_{f}=\frac{4F_{\max}}{\pi d^{2}}$$

where *F*_max_ is the fracture load, *d* is the fiber diameter. A minimum of twenty specimens were tested for each CF type.

The single-fiber fragmentation test was conducted using a universal tensile test machine (BTM5105, Shenyang, China) under a constant displacement rate of 0.5 mm/min. The fragmentation length was measured through a Zeiss Axio Scope A1 metalloscope (Oberkochen, Germany). The IFSS (*τ*) was determined based on the following equations [24,25]:

(2) $$\tau =\frac{\sigma_{f}}{2\left(\frac{l_{c}}{d}\right)}$$

(3) $$l_{c}=\frac{4}{3}\bar{l}$$

where *l_c_* is the critical length, $\bar{l}$ is the average of fragmentation length measured from the metalloscope.

The three-point short beam shear test was carried out to determine the ILSS of the unidirectional carbon fiber composites by following the ASTM D2344 standard [26]. The length, width, and thickness of the specimens were 24, 8, and 4 mm, respectively. The test was performed using a universal mechanical test machine (BTM5104, Shenyang, China) with a constant crosshead speed of 1 mm/min and a span width of 16 mm (span-to-thickness ratio was 4). The *ILSS* was calculated by the following equation [27,28]:

(4) $$ILSS=\frac{3P}{4bh}$$

where *P* is failure load observed during the test, and *b* and *h* are the specimen width and thickness, respectively. At least five specimen were tested for each composite. 

## 3. Results and Discussion

**Figure 1:**
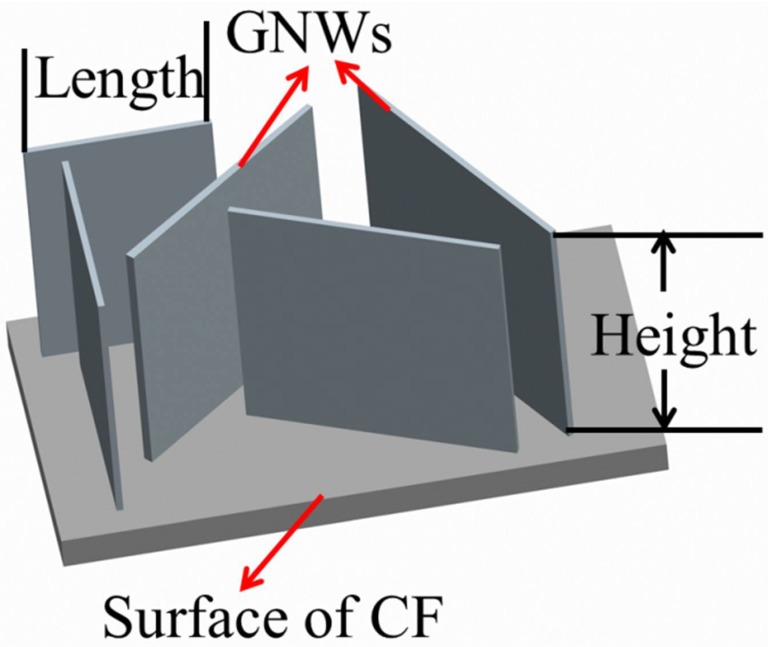
Simplified schematic illustration of graphene nanowalls on the surface of carbon fiber.

**Figure 2:**
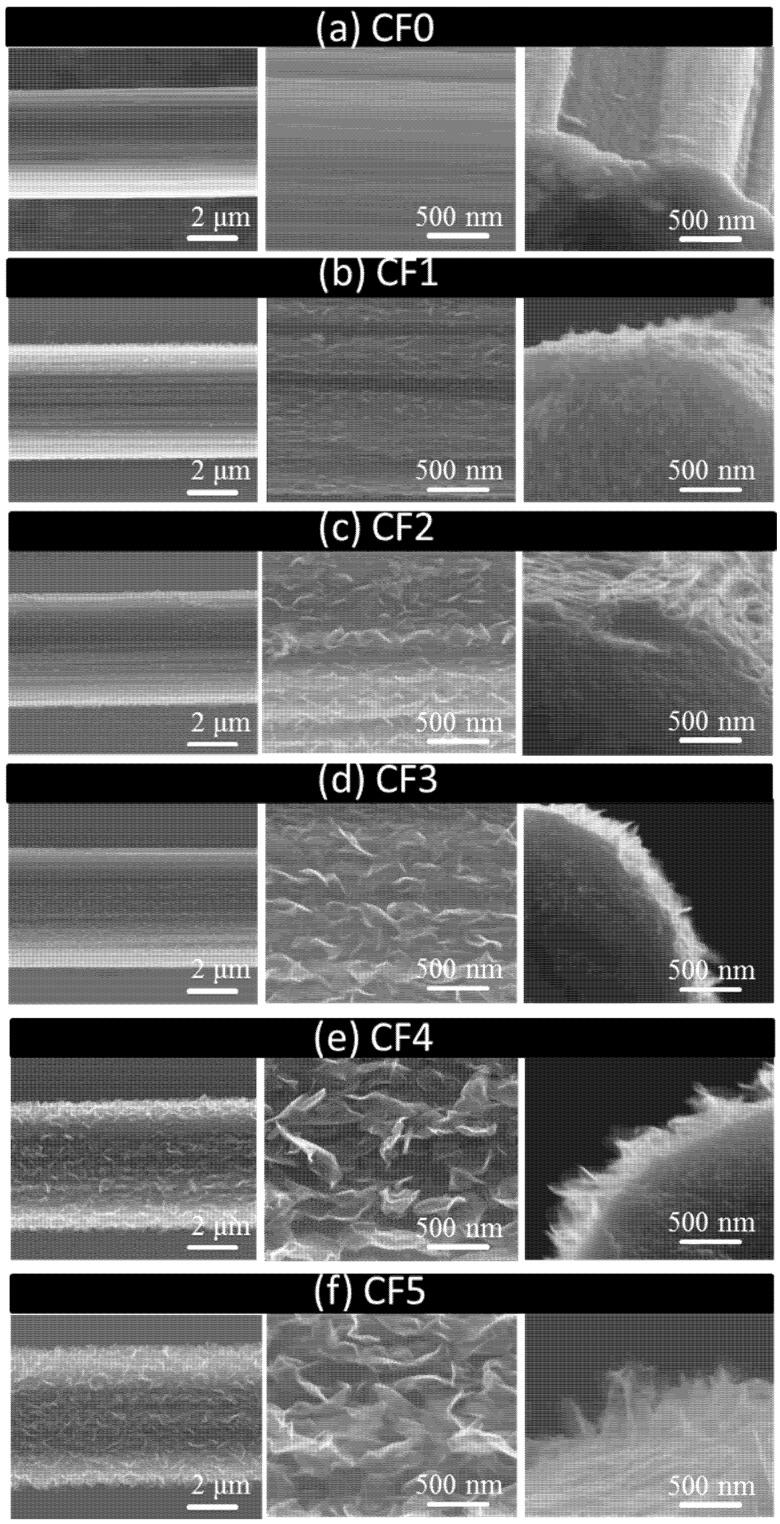
Surface and cross-sectional scanning electron microscope (SEM)images of (**a**) CF0; (**b**) CF1; (**c**) CF2; (**d**) CF3; (**e**) CF4 and (**f**) CF5.

**Figure 3:**
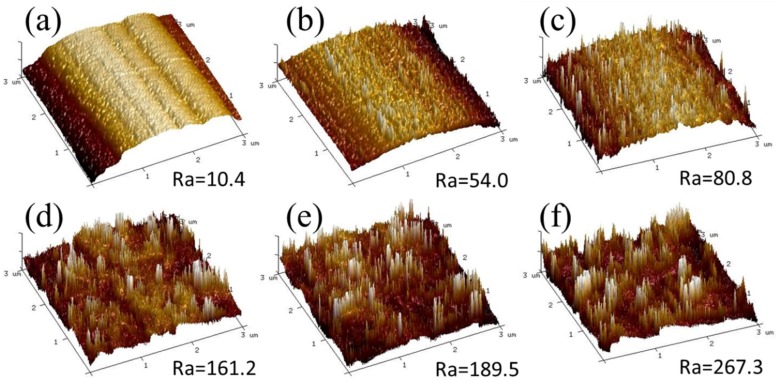
Atomic force microscope (AFM) surface images of (**a**) CF0; (**b**) CF1; (**c**) CF2; (**d**) CF3; (**e**) CF4 and (**f**) CF5.

RF-PECVD system provides a simple, effective, and catalyst-free approach to directly depositing high-quality GNWs on CF surfaces. Figure 1 shows a simplified illustration of the GNWs, indicating their size and layout on the substrate. The surface and cross-sectional morphologies of the GNW-modified CFs with different deposition time are shown in Figure 2. It was observed that the carbon fibers were coated by GNWs. The surface of the modified fibers became rougher compared to that of the unmodified fiber. It demonstrated the successful growth of GNWs on the CF surfaces. The size of GNWs for each fiber type is listed in Table 1. The result indicated that the length and height of the GNWs continuously increased with the deposition time. The AFM images of CFs are compared in Figure 3. The pure fiber surface displayed a few narrow grooves along the longitudinal way due to the chemical oxidation process during manufacturing. After depositing GNWs, the fiber surface including the grooves was covered by the nanowalls. The roughness of the surface significantly increased with the increase of the nanowall size. It was in accordance with the SEM results. We believe the increased GNW size and the surface roughness of CFs could play important roles in the interfacial shear strength of CFRCs, since they were closely related to the mechanical interlocking structure with the matrix. 

**Figure 4:**
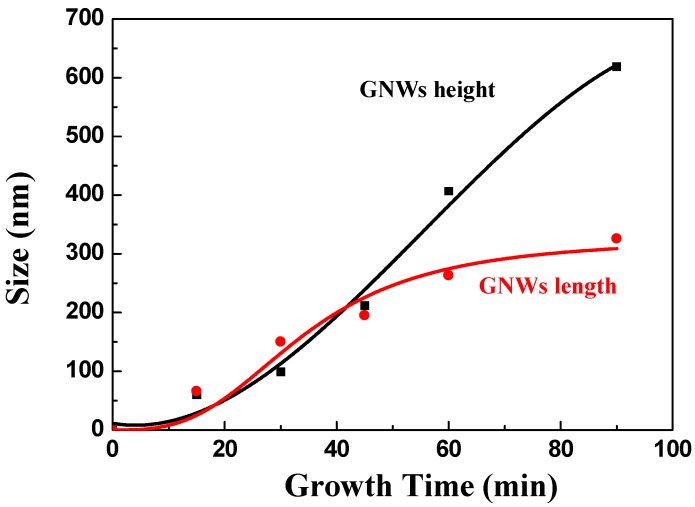
Length and height of graphene nanowalls (GNWs) versus growth time.

To further understand the primary deposition process of GNWs on CF, GNWs were simplified as regular-shaped nanowalls as illustrated in Figure 1, and the size parameters were plotted versus growth time in Figure 4. In the initial stage of the GNWs’ growth, a very thin amorphous carbon layer was formed on the CF surface through absorbing hydrocarbon radicals. Dangling bonds associated with nucleation sites were then produced, initiating the subsequent two-dimensional growth of graphene sheets, perpendicular to the substrate [29]. The growth rate of GNWs at this stage was low as suggested in Figure 4 in the first few minutes of the deposition. As growth period increased, the nanowalls continued to grow vertically and horizontally. The growth rates in both directions were comparable. The GNWs were nearly square-shaped for CF-1, CF-2, and CF-3. However, with the further increase of growth time after 45 min, spreading GNWs met one another in a horizontal direction, and then became saturated, resulting in the formation of maze-like linked nanowalls. Thus, the growth in this direction was confined, and the increase of the GNW length was limited. In contrast, the growth rate in the vertical way was almost unaffected. The height of the nanowalls continuously increased at a constant rate. As a result, the GNWs of CF4 and CF5 were nearly rectangular-shaped. 

The single-fiber tensile test was performed to investigate the effect of the CVD process on the mechanical property of the fiber. The tensile strength of the original CF (as received) was 3.9 ± 0.78 GPa. After removing the sizing agent, the strength decreased to 3.6 ± 0.65 GPa (CF0). It demonstrated the enhancement effect of the sizing agent in the fabrication of CF. Moreover, the tensile strength for each set of GNW-modified CF is shown in Table 1. The strength was slightly lower than that of CF0 without GNW coating. The CVD process for GNW growth at high temperature could produce defects on the fiber surface and then reduce the mechanical properties of the fiber. It should be noted that the strength firstly decreased then increased to a certain level with the increase of growth time. The recovery of the tensile strength implied that GNWs could bridge the surface defects of the raw fibers. 

**Figure 5:**
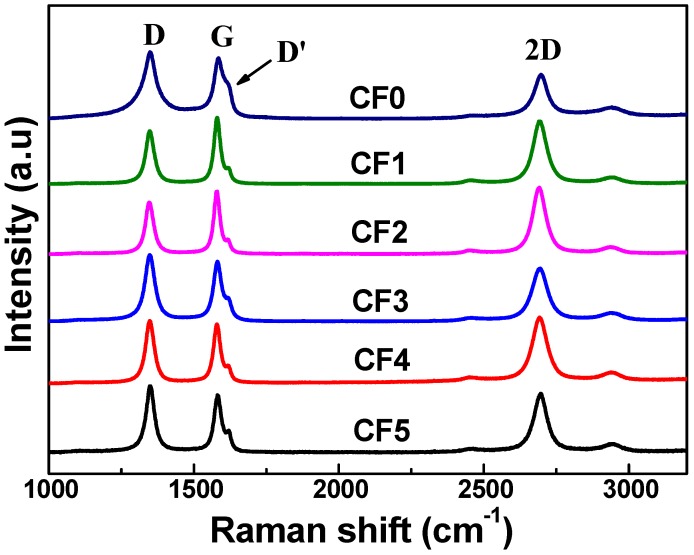
Raman spectra of CF0; CF1; CF2; CF3; CF4 and CF5.

Raman spectra of the GNW-modified CFs are presented in Figure 5. The spectrum of the pure carbon fiber is also shown as reference. For the GNW-modified CFs, they displayed a G-band at around 1580 cm^−1^ due to the in-phase stretching vibration of pairs of sp^2^ atoms in the graphene lattice. Distinguished D-band at 1350 cm^−1^ and D’-band at 1620 cm^−1^ corresponded to the lattice defects or crystal imperfections from different nature. The prominent 2D-band at 2700 cm^−1^ was known as the second-order of the D-band. In particular, the key spectral parameters are listed in Table 2. It can be observed that the GNWs with different sizes had similar *I*(D)*/I*(D’) ratios of about 3, which indicated the presence of boundary-like defects [30] in all GNWs. In contrast, the ratio of the pure CF was 1.71, which could be attributed to the original surface defects on the fiber. A noticeable change of defect type was observed after the deposition of the nanowalls. We believe the surface defects could strongly influence the tensile properties of the fibers, while the boundary defects between the GNWs could be more important in determining the interfacial strength. *I*(D)/*I*(G) ratio revealed the defects concentration of GNWs. It varied from 0.8 to 1.2 with the increase of nanowall size from CF1 to CF5. The bigger size of GNWs associated with longer plasma treatment could result in the increase of defects concentration in the nanowalls. A sharp increment of the ratio was noticed in CF3, where the growth of the nanowalls in a horizontal direction was confined and nearly saturated. It may significantly contribute to the formation of defects especially at the GNWs’ boundaries. Moreover, the *I*(G)*/I*(2D) ratio and full width at half maximum (FWHM) of 2D-band were measured to reveal the layer stacking characters of the nanowalls. The values of the ratio and FWHM for all the GNWs were about 1 and 55 cm^−1^, respectively. It implied that the GNWs could be composed of few stacked graphene layers with a similar layer number [20,31]. Therefore, the GNWs on CF1 to CF5 differed in their length and height, but had similar wall thickness.

**Figure 6:**
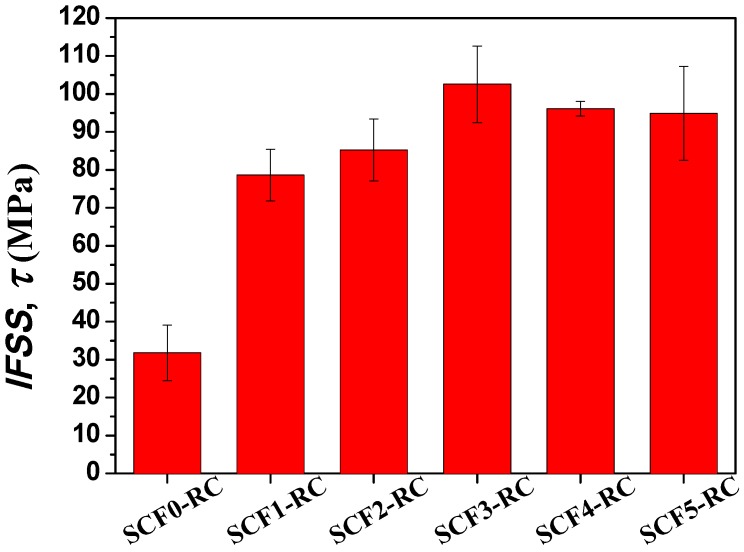
Interfacial shear strength of single carbon fiber composites. The error bars represent standard deviations.

Interfacial shear strength describes the ability of a CFRC to resist fiber debonding or adhesive failure that occurs in the interface. It is a vitally important property for design applications of CFRCs. The single-fiber fragmentation test is widely used to assess the shear strength at the fiber/matrix interface [13,32]. Figure 6 shows the average IFSS values of the CF-reinforced composites that contained different sizes of GNWs. The IFSS of the CF0-RC without GNWs was 31.78 MPa. In comparison, the GNW-modified composites exhibited significantly higher interfacial shear strength compared to the unmodified composite, although the strength of the fibers slightly reduced after the CVD process. The presence of GNWs substantially increased the surface roughness of the carbon fiber, providing massive ‘tenon-mortise’-like mechanical interlocks in the CFs/epoxy interface in a large area. Thus, the interlocks effectively enhanced the interfacial shear strength. The IFSS value increased with the increase of the GNW size and reached to 102.57 MPa for SCF3-RC, which corresponded to a 222.8% increase in the interfacial shear strength. In contrast, modification of the CF/epoxy interface by carbon black, CNTs, GO, and ZnO increased the IFSS of the composites by 44.4% [27], 89.4% [28], 75.6% [33], and 113% [13], respectively. GNWs were superior to other nanomaterials in reinforcing IFSS. However, it was noted that as the GNW size further increased, the IFSS slightly decreased to 96.11 and 94.86 MPa when CF4 and CF5 were deposited on the CF surface, respectively. The enhancement ability of the nanowalls in IFSS became weaker. Such a phenomenon occurred when the shape of the nanowalls changed from square to rectangle. The growth of the nanowalls in a horizontal direction was nearly saturated. Although the increase in GNW height could provide more interfacial interlocks for enhancement, the defect concentration at the GNWs’ boundaries simultaneously increased and deteriorated the interfacial shear strength. Moreover, as shown in Figure 2, the graphene nanowalls’ surface exhibited a wrinkle-like texture. It has been reported that a bigger size of graphene nanoplatelets could possess larger size wrinkles, and the size-increased wrinkles may reduce the load transfer efficiency in graphene layers and cause local stress concentration of the composites [34]. Hence, the reinforcement of IFSS was compromised for SCF4-RC and SCF5-RC. 

**Figure 7:**
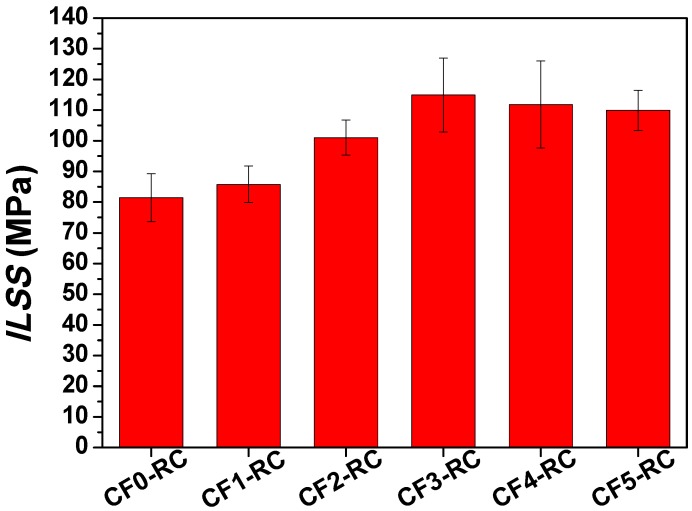
Interlaminar shear strength of unidirectional carbon fiber laminate composites. The error bars represent standard deviations.

Interlaminar shear strength (ILSS) was examined to evaluate the delamination resistance and bond strength of the composites. The failure behaviour was closely related to the resin deformation and crack propagation. Figure 7 presents the ILSS values of the composites with and without GNWs. It revealed that the incorporation of the nanowalls showed a similar effect on the interlaminar shear strength as that which was observed in the IFSS test. Specifically, the ILSS of CF0-RC was 81.46 MPa. By depositing GNWs on the CF surface, the composites possessed higher ILSS compared to that of the unmodified composite. It was attributed to the strong mechanical interlocking at the interface and the improved wettability of resin on the fiber surface. The introduction of GNWs could effectively transfer load from the matrix to the fiber. The toughness and strength of the interfacial area surrounding the CF were enhanced, which then resulted in the improved resistance to crack propagation and opening. CF3-RC was measured to have the maximum interlaminar shear strength of 114.93 MPa with an enhancement of 41.1%, which was at a high level of improvement for CFRC in the literatures [15,27,35]. However, further increase of GNW size led to no more improvement in ILSS. The increased defect concentration and wrinkle size could be responsible for the weakened properties. 

**Figure 8:**
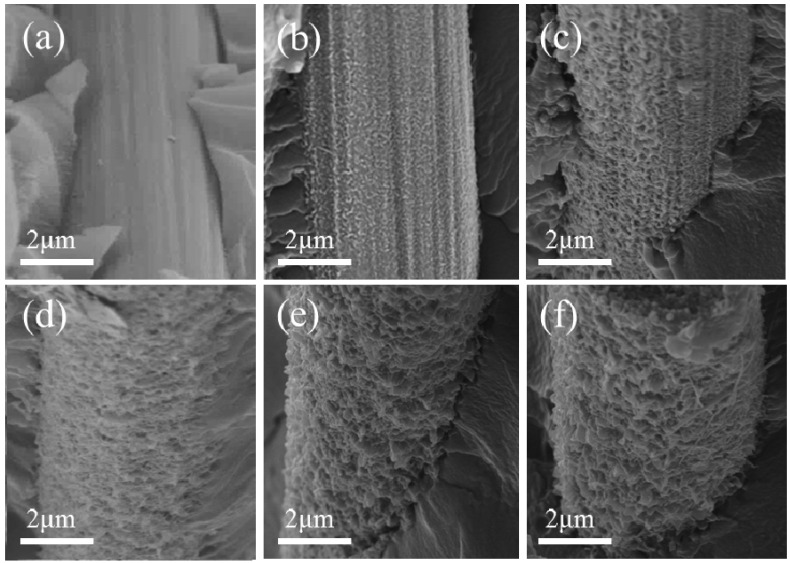
SEM images of the fracture surface for (**a**) CF0-RC; (**b**) CF1-RC; (**c**) CF2-RC; (**d**) CF3-RC; (**e**) CF4-RC and (**f**) CF5-RC.

The reinforcement mechanism was further understood by SEM fractography analysis. The morphologies of the fracture surfaces for the CFRC are depicted in Figure 8. It was noticed in Figure 8a that the carbon fiber without GNWs showed a smooth and clear surface. The resin completely detached from the fiber after fracture because of the poor wettability and adhesion with the resin matrix. The weak interface could hardly facilitate load transfer between the CF and the matrix, which led to a rapid crack propagation followed by a complete interfacial debonding. A typical brittle-fractured morphology was observed in the composite. Therefore, the measured ILSS value was low. In comparison, the composites embedded with GNW-modified CFs exhibited significantly different fracture surfaces as shown in Figure 8b–f. The resin was well penetrated into the nanowalls, forming a strong mechanical interlocking structure in the CF/matrix interface. The GNWs acted as physical links that substantially elevated the interfacial adhesions, which enabled effective load transfer. On the other hand, the deposition of the nanowalls substantially increased the surface roughness of the fibers. It generated a large amount of fracture surface area at the interface, resulting in greater energy absorption when fractures occurred. Thus, the fracture toughness was elevated as compared to the unmodified fiber-embedded composite. Furthermore, a number of leaf-like fracture patterns appeared on the fracture surfaces of the composites with GNWs. It indicated that the presence of the nanowalls effectively resisted the propagation of cracks by producing a considerable plastic deformation. The enhancement effect of GNWs on the interfacial strength of CFRC and the corresponding mechanisms were again revealed. 

## 4. Conclusions 

In summary, GNWs with different sizes were firmly coated on the surface of CFs by means of the RF-PECVD method. The size of the GNWs was closely related to the growth time during the deposition process. The height of GNWs continuously increased and exhibited a linear relationship with the growth time. In contrast, the increase of the GNW length was limited after 45 min. The growth of nanowalls in a horizontal direction became nearly saturated. Raman spectrum revealed that boundary-like defects existed in the GNWs and the defect concentration increased with the increase of the GNW size. The nanowalls varied in size but may possess similar wall thickness. According to the single-fiber fragmentation test, the IFSS value of the CFRC without GNWs was 31.78 MPa. The incorporation of GNWs significantly increased the interfacial shear strength, and the value reached 102.57 Mpa for the composite with CF3, which corresponded to a 222.8% enhancement of IFSS. On the other hand, the results of the three-point short beam shear test demonstrated a 41.1% increase of ILSS after introducing the same type of carbon fiber. The reinforcement mechanism was understood based upon observation and analysis of the fracture surfaces of composites. GNWs acted as physical links that provided strong mechanical interlocking in the CF/matrix interface. However, further increasing the GNW size could not lead to more improvement in IFSS and ILSS. The increased wrinkle size and defect concentration in the nanowalls could deteriorate the mechanical properties. 

## Figures and Tables

**Table 1:** Properties of graphene nanowalls (GNW)-modified carbon fibers (CFs).

Parameter	CF-0	CF-1	CF-2	CF-3	CF-4	CF-5
Growth time (min)	0	15	30	45	60	90
GNW length (nm)	0	66	150	195	263	326
GNW height (nm)	0	60	99	212	407	618
*σ_f_* (GPa)	3.60 ± 0.65	3.26 ± 0.64	3.31 ± 0.68	3.41 ± 0.74	3.48 ± 0.64	3.41 ± 0.69

**Table 2:** Key Raman spectral parameter.

Parameter	CF-0	CF-1	CF-2	CF-3	CF-4	CF-5
*I*(D)*/I*(D’)	1.71	3.05	3.20	2.93	3.12	2.98
*I*(D)*/I*(G)	1.09	0.80	0.82	1.08	1.11	1.20
*I*(G)/*I*(2D)	1.20	1.05	0.97	1.09	0.94	0.96
FWHM (2D)(cm^−1^)	55.5	54.7	52.9	56.3	57.4	53.5
